# Iris metastasis from lung adenocarcinoma diagnosed solely through aqueous humor cytology: A case report

**DOI:** 10.1016/j.ajoc.2026.102605

**Published:** 2026-05-25

**Authors:** Atsushi Okubo, Satoru Kanda, Kimiko Okinaga, Kiyoshi Ishii, Shuichiro Aoki, Kohdai Kitamoto, Ryo Terao, Keiko Azuma

**Affiliations:** a Department of Ophthalmology, The University of Tokyo, 7-3-1 Hongo, Bunkyo-ku, Tokyo, 113-8655, Japan; b Department of Ophthalmology, Japanese Red Cross Saitama Hospital, 1-5 Shintoshin, Chuo-ku, Saitama City, Saitama Prefecture, Japan

**Keywords:** Adenocarcinoma, Aqueous paracentesis, Cytology, EGFR, Iris, Metastasis, Oncology

## Abstract

**Purpose:**

We report a unique case of iris metastasis from epidermal growth factor receptor (EGFR)-positive lung adenocarcinoma, diagnosed solely by cytologic analysis of aqueous humor without tissue biopsy.

**Observations:**

A 56-year-old man with a known history of lung adenocarcinoma presented with unilateral iris masses, anterior chamber inflammation, and secondary glaucoma. Intraocular pressure was initially controlled with cataract surgery combined with microhook trabeculotomy. However, the pressure re-elevated despite maximal topical antiglaucoma therapy (including a prostaglandin analog, beta-blocker, carbonic anhydrase inhibitor, alpha-2 agonist, and Rho-kinase inhibitor) and oral acetazolamide. Three sessions of transscleral cyclophotocoagulation were performed. In addition, two intravitreal injections of aflibercept (2.0 mg each) were administered for rubeosis iridis. Aqueous humor obtained via anterior chamber paracentesis immediately before intravitreal injection demonstrated Class V adenocarcinoma cells on cytology, allowing the diagnosis of iris metastasis without iris biopsy.

The lesions subsequently enlarged, and similar iris lesions appeared in the fellow eye during the final 1 month before the patient's death. Systemic disease progression became evident during the final 1 month before death, and the patient ultimately succumbed 9 months after the initial visit. Conclusions and Importance: This is among the few reported cases of iris metastasis confirmed solely by aqueous cytology. This case underscores the diagnostic utility of anterior chamber paracentesis as a less invasive yet reliable alternative to iris biopsy in carefully selected patients. It also highlights the clinical course and therapeutic challenges in managing anterior segment metastasis from epithelial tumors such as lung cancer.

## Highlights

• Iris metastasis may mimic anterior uveitis or secondary glaucoma in cancer patients.
• Aqueous humor cytology enabled biopsy-free diagnosis of iris metastasis.
• Class V aqueous cytology supported metastatic adenocarcinoma in a patient with epidermal growth factor receptor-mutant non–small cell lung cancer.
• Aqueous humor cytology supported biopsy-free confirmation during longitudinal management of tumor-associated secondary glaucoma.

**Claim of priority**

After conducting a literature review on October 27, 2025, utilizing PubMed, Google Scholar, and Ichushi-Web using the key words “iris metastasis,” “aqueous humor cytology,” and “lung adenocarcinoma,” we did not find any prior reports of iris metastasis diagnosed solely based on aqueous humor cytology from lung adenocarcinoma without tissue biopsy confirmation.

## 1 Introduction

Iris tumors comprise a diverse group of lesions ranging from benign nevi and cysts to malignant melanomas and metastatic deposits.^1^ Among these, metastatic iris tumors are rare but are clinically significant because they can mimic inflammatory or neovascular processes.^2^ Recognizing their characteristics and establishing a timely diagnosis is vital for appropriate clinical management.^3^

Whereas the uveal tract is a common site for ocular metastasis due to its rich vascular supply, most intraocular metastases involve the choroid, whereas the iris accounts for only 8%–11% of cases.^4^^,^^5^ The iris and ciliary body, which constitute the anterior uveal tract, are affected less frequently. Lung and breast cancers are the most common primary malignancies that metastasize to the iris, with lung adenocarcinoma comprising a substantial proportion.^6^^,^^7^

Diagnosing iris metastasis is challenging because of its diverse clinical presentation. Patients may exhibit symptoms like blurred vision, photophobia, elevated intraocular pressure (IOP), iris neovascularization, or signs that mimic anterior uveitis or neovascular glaucoma.^2^^,^^6^ These presentations often lead to misdiagnosis, especially in patients without a known history of systemic cancer. However, even in patients with a confirmed malignancy, the rarity and subtlety of iris metastasis can delay an accurate diagnosis.

Reports in which aqueous humor cytology alone establishes a definitive diagnosis of iris metastasis without tissue sampling are limited. In addition, ocular metastasis is commonly detected in the setting of systemic disease progression, and anterior segment involvement can pose diagnostic uncertainty when systemic disease appears clinically stable.

Here, we describe a case of iris metastasis from lung adenocarcinoma in which the diagnosis was established by aqueous humor cytology (Class V) without iris biopsy. At the time of ocular presentation, systemic evaluation showed no new or progressive metastatic lesions. This report links biopsy-free cytologic confirmation to a time-stamped clinical course of tumor-associated secondary glaucoma management and follow-up findings, including subsequent contralateral iris involvement.

## 2 Case presentation

A 56-year-old man with epidermal growth factor receptor (EGFR)-mutant lung adenocarcinoma, previously treated with systemic therapy (osimertinib), was referred to our department for right eye symptoms, including decreased vision and elevated IOP.

**Fig. 1:**
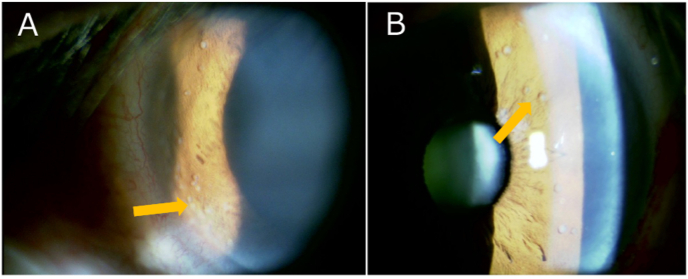
Slit-lamp photographs of a 56-year-old man with epidermal growth factor receptor-mutant lung adenocarcinoma and iris metastases. (A) Right eye at presentation showed multiple elevated whitish iris nodules (arrow). (B) Left eye at follow-up (8 months after presentation; 1 month before death) showed multiple elevated white iris nodules (arrow), consistent with contralateral iris involvement.

Upon initial examination, the best-corrected visual acuity was 0.3 in the right eye and 1.2 in the left eye. Although the right pupil was fixed and the view was limited by corneal edema, relative afferent pupillary defect was assessed by reverse testing (observing the left pupillary response) and was not detected. The right eye exhibited a markedly elevated IOP of 48 mmHg (vs. 15 mmHg in the left), fixed mydriasis, corneal edema, and anterior chamber inflammation with 2+ cells and 2+ flare, as well as multiple elevated white nodules on the iris surface (Fig. 1A). The fundus details were partially obscured in the right eye due to corneal edema, but there were no apparent signs of retinal or choroidal involvement in both eyes. The fixed dilated pupil in the right eye was thought to result from tumor infiltration or iris sphincter dysfunction.

**Fig. 2:**
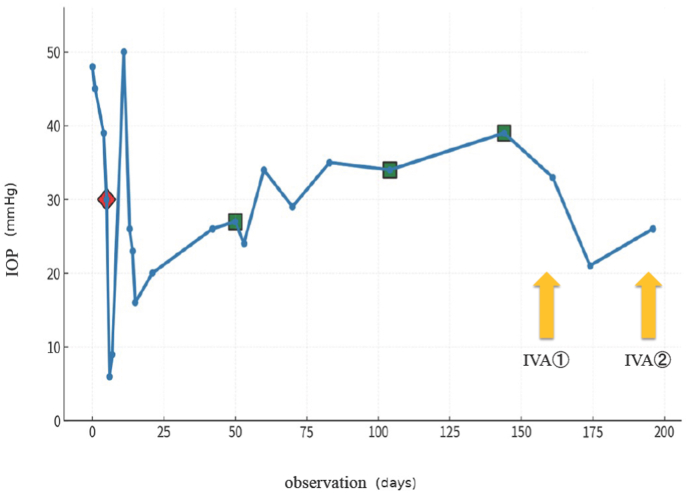
Timeline of serial intraocular pressure (IOP) measurements in the right eye of a 56-year-old man with epidermal growth factor receptor-mutant lung adenocarcinoma and iris metastases, from the initial visit (Day 0). The red diamond (◆) indicates the timing of combined phacoemulsification and microhook trabeculotomy (Day 5). Green squares (■) indicate sessions of transscleral cyclophotocoagulation performed (IRIDEX Cyclo G6® Glaucoma Laser System) on Days 50, 104, and 144. Arrows indicate two intravitreal aflibercept injections (IVA; 2.0 mg each) administered for rubeosis iridis (Day 161 and 196). (For interpretation of the references to colour in this figure legend, the reader is referred to the Web version of this article.)

IOP was initially managed with topical antiglaucoma medications (including a prostaglandin analog, beta-blocker, carbonic anhydrase inhibitor, alpha-2 agonist, and Rho-kinase inhibitor) and oral acetazolamide. Five days after initial presentation, the patient underwent combined phacoemulsification with intraocular lens implantation and microhook trabeculotomy, resulting in transient IOP control (range, 6-10 mmHg). However, the IOP gradually increased to 50 mmHg despite maximal topical antiglaucoma therapy (five classes: a prostaglandin analog, beta-blocker, carbonic anhydrase inhibitor, alpha-2 agonist, and Rho-kinase inhibitor) and oral acetazolamide, prompting three sessions of transscleral cyclophotocoagulation (TS-CPC). TS-CPC was performed in three sessions for recurrent IOP elevation at days 50, 104, and 144 after initial presentation. At each session, laser treatment was applied from 4 to 8 o'clock inferiorly and from 10 to 2 o'clock superiorly (total 8 clock hours), sparing the 3 and 9 o'clock meridians, at 2500 mW, with 80 s delivered to each arc (total 160 s per session). During follow-up, rubeosis iridis developed in the right eye; therefore, two intravitreal injections of aflibercept (2.0 mg each) were administered to treat iris neovascularization in the right eye. The second injection was given 35 days after the first because neovascularization persisted. The longitudinal IOP trend and timing of interventions are shown in Fig. 2.

**Fig. 3:**
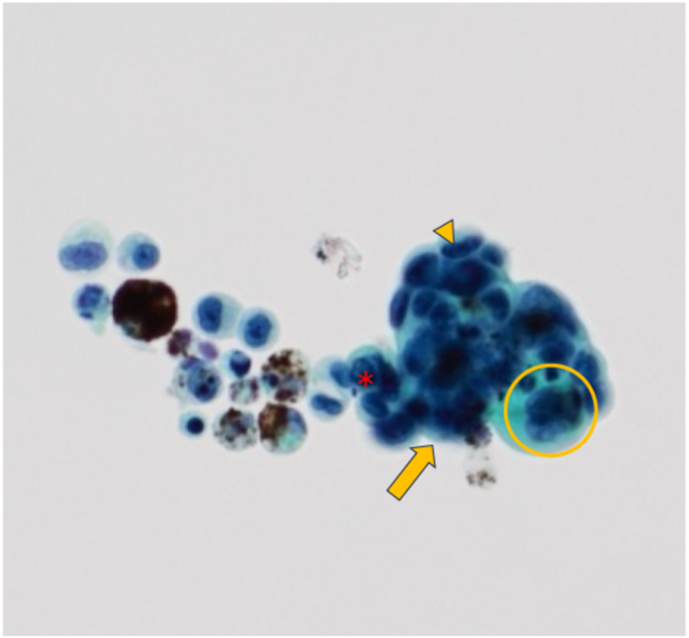
Aqueous humor cytology from a 56-year-old man with epidermal growth factor receptor–mutant lung adenocarcinoma and multiple elevated whitish iris nodules in the right eye. Clusters (arrow) and scattered atypical glandular epithelial cells (∗) with pleomorphic nuclei (arrowhead) and prominent nucleoli (open circle) are present, consistent with metastatic adenocarcinoma. (H&E stain, ×400).

Cytological analysis of the aqueous humor sample, obtained by anterior chamber paracentesis immediately before the second intravitreal aflibercept injection, revealed many cohesive clusters and isolated atypical glandular epithelial cells, characterized by marked nuclear pleomorphism, prominent nucleoli, and evidence of glandular differentiation (Class V) (Fig. 3). These cytomorphological features are notable indicators of metastatic adenocarcinoma. Considering the patient's clinical history of EGFR-mutant lung adenocarcinoma and the absence of other clinically evident progressive visceral metastases at that time, the diagnosis of iris metastasis was established based on aqueous humor cytology alone. Following the cytologic findings, systemic restaging was performed, including contrast-enhanced computed tomography of the chest and abdomen and brain magnetic resonance imaging, which demonstrated multiple bone metastases in the spine, sacrum, pelvis, bilateral humeri, bilateral femora, bilateral scapulae, and sternum. Notably, this case demonstrates that anterior chamber paracentesis can yield diagnostically sufficient cytological material even without an iris biopsy, offering a minimally invasive alternative in selected cases.

Subsequent management was focused on preserving vision and ocular comfort. As the iris tumors demonstrated slow progression and the patient's visual acuity and symptoms remained stable, enucleation was not required.

However, approximately 8 months after the initial visit, similar iris lesions with multiple white nodules developed on the surface of the temporal iris in the left eye (Fig. 1B). This finding was consistent with bilateral metastatic involvement of the iris. Ultimately, 9 months after the initial visit, despite ongoing therapy, the patient died from systemic progression of lung cancer.

## 3 Discussion

We report a case of iris metastases originating from EGFR-mutant lung adenocarcinoma, in which the diagnosis was established by cytologic analysis of aqueous humor without iris biopsy. This case highlights the diagnostic value of anterior chamber paracentesis as a less invasive yet reliable approach when tissue sampling is undesirable or clinically challenging.

Among pulmonary malignancies, adenocarcinoma is the most common histologic subtype involved in iris metastasis, especially in EGFR-mutant tumors treated with tyrosine kinase inhibitors (e.g. osimertinib).^8^ Several anatomical and physiological factors may explain the relative infrequency of iris and ciliary body metastases compared to choroidal involvement. First, the iris has a significantly lower vascular density than the choroid, reducing the likelihood of hematogenous seeding.^9^ Second, the iris is primarily composed of muscular tissue, which may represent a less favorable microenvironment for tumor cell colonization. Third, the constant contractile motion of the iris may inhibit metastatic cell adhesion and establishment.^9^

In most published cases, obtaining a definitive diagnosis has necessitated sampling of iris tissue via fine-needle aspiration, iridectomy, or biopsy to distinguish metastasis from primary iris tumors or granulomatous disease.^4^^,^^10^^,^^11^ Ciftci et al.^12^ reported bilateral iris metastases from small-cell lung carcinoma confirmed by aspiration biopsy, whereas Goto et al.^13^ confirmed iris metastasis from small-cell lung carcinoma by anterior chamber fluid cytology. Goto et al. also reported intravitreal aflibercept treatment for associated iris neovascularization and neovascular glaucoma.^13^ Konno et al. combined cytologic and aqueous humor tumor marker analyses to diagnose iris metastasis originating from lung adenocarcinoma, although a biopsy remained essential.^14^

Previous reports have also described the diagnostic utility of cytologic and fine-needle techniques for metastatic iris tumors. For example, Han et al.^15^ reported iris metastasis from non-small-cell lung cancer confirmed by repeated aqueous humor cytology. In contrast, Moon et al.^16^ described a case in which aqueous cytology was negative and the diagnosis was ultimately confirmed by iris biopsy. Takahashi et al. reported classic cases in which aqueous humor cytology was useful for anterior segment metastases.^17^ Other reports have utilized fine-needle aspiration cytology in metastatic iris tumors derived from lung adenocarcinoma^18^ and classic aqueous humor paracentesis for anterior segment metastases.^19^ While these studies support the feasibility of cytology-based diagnosis, they often provide limited detail regarding time-stamped glaucoma management and longitudinal IOP behavior in tumor-associated secondary glaucoma.

In contrast, our report links cytologic confirmation directly to clinical decision-making without iris biopsy and provides a granular, day-by-day treatment timeline with serial IOP measurements, including maximal topical therapy with oral acetazolamide, combined phacoemulsification with microhook trabeculotomy, three sessions of TS-CPC, and intravitreal aflibercept for rubeosis iridis (Fig. 2). Furthermore, we document contralateral iris involvement during the 1 month before death and the systemic outcome over follow-up, completing the clinical narrative from presentation to terminal progression. These elements complement prior reports and help clarify clinical scenarios in which aqueous humor cytology may serve as a practical, minimally invasive diagnostic alternative to tissue biopsy in anterior segment masquerade presentations (Table 1).

**Table 1:** Comparison of the present case with selected reports of iris metastasis and cytology-based diagnosis.

Study	Age/Sex	Primary cancer	Diagnostic confirmation	Aqueous humor role	Iris biopsy required	Key ocular features
Shields et al., 1997^4^	Series	Various systemic cancers (lung/breast common)	Clinical ± histopathology (series)	Not reported	Variable (FNAB/biopsy in some)	Iris lesions with uveitis-like findings; secondary glaucoma (series)
Wong et al., 2022^8^	81/F	EGFR-mutant lung adenocarcinoma	Clinical diagnosis in known cancer setting	Not used as primary diagnostic method	No (biopsy performed later to confirm tumor regression)	Iris metastasis treated with osimertinib + monthly intravitreal bevacizumab; complete regression
Hernández-Da Mota et al., 2018^10^	76/M	Lung adenocarcinoma	Histopathology (iris tissue sampling)	Not reported	Yes	Iris metastasis as first manifestation; anterior segment findings
Mitamura et al., 2025^11^	80/F	Cecal adenocarcinoma	Fine-needle aspiration cytology (iris mass) plus aqueous humor tumor marker (carcinoembryonic antigen)	Supportive/contributory (tumor marker assay)	Yes (FNAB cytology)	Iris mass; diagnosis supported by markedly elevated aqueous carcinoembryonic antigen; treated with ocular irradiation
Ciftci et al., 2024^12^	46/M	Small cell lung carcinoma	Aspiration biopsy/FNAB histopathology	NR	Yes	Bilateral vascularized iris masses; no secondary glaucoma; IOP 12 mmHg both eyes
Goto et al., 2025^13^	65/M	Small cell lung carcinoma	Aqueous humor cytology (anterior chamber fluid cytology)	Diagnostic/Confirmatory	NR	Anterior chamber cells, multiple grayish-white iris masses, iris neovascularization, neovascular glaucoma, IOP 55 mmHg; treated with intravitreal aflibercept
Konno et al., 2024^14^	73/M	Lung adenocarcinoma	Iris biopsy	Elevated aqueous carcinoembryonic antigen supported suspicion	Yes	Masquerade-type anterior segment findings
Han et al., 2023^15^	53/F	Non–small cell lung cancer	Repeat aqueous humor cytology ± histopathologic confirmation	Diagnostic (repeat cytology)	NR/histopathologic confirmation reported	Iris metastasis with anterior segment inflammation and elevated IOP (details NR)
Moon et al., 2024^16^	63/F	Non–small cell lung cancer	Iris biopsy (histopathology)	Performed; cytology negative	Yes	Anterior uveitis + secondary glaucoma; multiple iris nodules; IOP 52 mmHg
Takahashi et al., 1984^17^	3 cases	Metastatic carcinoma to iris/ciliary body (includes lung adenocarcinoma)	Aqueous humor cytology in 2 cases; iris biopsy during trabeculectomy in 1 case	Diagnostic (2/3 cases)	Yes (1/3 cases)	Refractory iridocyclitis-like presentation with secondary glaucoma; iris/angle nodules
Mitamura et al., 2020^18^	56/F	Lung adenocarcinoma	Fine-needle aspiration cytology (iris mass)	NR	Yes (FNAB cytology)	Uveitis with a 2×2-mm inferior iris mass; pain/redness; normal IOP; AS-OCT monitoring before/after radiotherapy
Scholz et al., 1983^19^	63/F; 34/F	Small-cell lung carcinoma; breast adenocarcinoma	Aqueous paracentesis/cytopathologic examination	Diagnostic	No ocular tissue biopsy	Iris nodules or whitish chamber-angle mass; anterior segment involvement mimicking uveitis; treated with irradiation and/or chemotherapy
Present case	56/M	EGFR-mutant lung adenocarcinoma	Aqueous humor cytology (Class V)	Definitive diagnosis	No	Fixed mydriasis; multiple iris nodules; secondary glaucoma; rubeosis iridis; treated with combined phacoemulsification and microhook trabeculotomy, transscleral cyclophotocoagulation and intravitreal aflibercept

**Abbreviations:** AS-OCT, anterior segment optical coherence tomography; EGFR, epidermal growth factor receptor; F, female; FNAB, fine-needle aspiration biopsy; IOP, intraocular pressure; M, male; NR, not reported.

The usefulness of aqueous humor cytology in anterior segment tumors, especially in epithelial metastases like lung adenocarcinoma, remains underreported. Whereas aqueous humor cytology is established for lymphoid and retinoblastoma diagnoses, its application in epithelial tumor metastases (such as lung adenocarcinoma) remains rarely reported.^20^^,^^21^ Our case contributes to the growing body of evidence supporting aqueous cytology as a minimally invasive and reliable diagnostic modality for certain anterior uveal metastases, particularly when exfoliated tumor cells are observed in the aqueous humor.

In this case, longitudinal management with cataract surgery, microhook trabeculotomy, and TS-CPC helped control IOP and preserve ocular comfort, while aqueous humor cytology enabled biopsy-free diagnostic confirmation. As the iris masses progressed slowly, visual function and comfort were preserved, and enucleation was avoided. This outcome highlights the importance of considering quality of life in patients with terminal systemic disease.

Several limitations should be acknowledged. Aqueous humor cytology may yield false-negative results depending on tumor burden, sampling volume, and the degree of tumor cell exfoliation; therefore, a negative cytology result does not exclude metastatic disease. When clinical suspicion remains high—particularly in patients with iris nodules, refractory inflammation, or secondary glaucoma—repeat sampling, iris tissue biopsy, and systemic restaging should be considered.

Finally, we recommend heightened clinical vigilance when evaluating cancer patients with refractory uveitis or secondary glaucoma. Cytologic analysis of the aqueous humor can serve as a valuable diagnostic alternative to biopsy and should be considered in appropriate clinical contexts. Larger case series and prospective studies are warranted to determine the diagnostic sensitivity and specificity of this approach in epithelial malignancies.

## CRediT authorship contribution statement

**Atsushi Okubo:** Writing – original draft. **Satoru Kanda:** Investigation. **Kimiko Okinaga:** Investigation. **Kiyoshi Ishii:** Investigation, Funding acquisition. **Shuichiro Aoki:** Writing – review & editing. **Kohdai Kitamoto:** Writing – review & editing. **Ryo Terao:** Writing – review & editing. **Keiko Azuma:** Writing – review & editing, Writing – original draft, Visualization, Validation, Supervision, Resources, Project administration, Methodology, Investigation, Conceptualization.

## Process

No datasets were generated or analyzed during the current study.

## Data statement

All data generated or analyzed during this study are included in this published article. Further inquiries can be directed to the corresponding author.

## Declaration of generative AI and AI-assisted technologies in the manuscript preparation

No generative AI or AI-assisted technologies were used in the writing of this manuscript.

## Funding

This research did not receive any specific grant from funding agencies in the public, commercial, or not-for-profit sectors.

## Declaration of competing interest

The authors declare that they have no known competing financial interests or personal relationships that could have appeared to influence the work reported in this paper.
